# Bipedal Walking of Underwater Soft Robot Based on Data-Driven Model Inspired by Octopus

**DOI:** 10.3389/frobt.2022.815435

**Published:** 2022-04-20

**Authors:** Qiuxuan Wu, Yan Wu, Xiaochen Yang, Botao Zhang, Jian Wang, Sergey A Chepinskiy, Anton A Zhilenkov

**Affiliations:** ^1^ Institute of Electrical Engineering, School of Automation, Hangzhou Dianzi University, Hangzhou, China; ^2^ HDU-ITMO Joint Institute, Hangzhou Dianzi University, Hangzhou, China; ^3^ Institute of Hydrodynamics and Control Processes, Saint-Petersburg State Marine Technical University, Saint Petersburg, Russia; ^4^ Faculty of Control Systems and Robotics, ITMO University, Saint Petersburg, Russia

**Keywords:** Octopus’s tentacle, soft arm, cable drive, data-driven model, deep reinforcement learning, bipedal coordinated walking

## Abstract

The soft organisms in nature have always been a source of inspiration for the design of soft arms and this paper draws inspiration from the octopus’s tentacle, aiming at a soft robot for moving flexibly in three-dimensional space. In the paper, combined with the characteristics of an octopus’s tentacle, a cable-driven soft arm is designed and fabricated, which can motion flexibly in three-dimensional space. Based on the TensorFlow framework, a data-driven model is established, and the data-driven model is trained using deep reinforcement learning strategy to realize posture control of a single soft arm. Finally, two trained soft arms are assembled into an octopus-inspired biped walking robot, which can go forward and turn around. Experimental analysis shows that the robot can achieve an average speed of 7.78 cm/s, and the maximum instantaneous speed can reach 12.8 cm/s.

## Introduction

With the development of ocean exploration and application, it is easy to cause damage to the target object and the environment when performing interactive operations on the marine environment, such as monitoring, biological sampling, seafloor landform and resource surveys, fishing for marine organisms and valuables, and maintaining marine underwater devices (Santina et al., 2018; Sinatra et al., 2019). Most autonomous robots with motion and operation functions are rigid, light-weight manipulators or claws for underwater transportation, and they are mainly operated for rough operations (Satja et al., 2018). Existing technology cannot cope with the vast and harsh environments that need monitoring and sampling the most. Compared with rigid and multi-joint robots, soft robots have continuous flexible deformation and manipulation capabilities, which are closer to biological softness. Given this, soft robots are used to aid in addressing the challenges posed by abyssal and wave-dominated environments (Aracri et al., 2021).

The appearance and movement characteristics of soft organisms in nature have always been a source of inspiration for the design of sophisticated soft arms (Nesher et al., 2020). When scientists studied the octopus’s tentacle, it was found that their taper angles range from 3° to 13.5°. A soft arm with a smaller taper angle can be bent into a larger curved shape, which can grasp lightweight items with a higher curvature more easily. A soft arm with a larger taper angle has a relatively smaller degree of bending and can grasp heavier and larger items with a lower curvature more easily (Xie et al., 2020).

The driving modes of soft manipulator are divided into cable drive, fibre-reinforced actuator, fluid-elastic pneumatic drive, variable stiffness pneumatic drive and intelligent biomimetic materials (Lu et al., 2020). The method of cable driven is easy to implement, easy to control and can transmit power over a long distance, and the inertia is also small, so this method is used to design the soft arm in this paper. There are two commonly used cable-driven methods including 4-cable drive and 3-cable drive, both of which can realize the three-dimensional drive of the soft arm. The driving cables of the former are symmetrically distributed in the soft arm at 90°, and the driving cables of the latter are symmetrically distributed in the soft arm at 120°. Because the 4-cable drive has an extra driving cable, theoretically the bearing capacity is greater, and the control will be more accurate. However, the 4-cable drive adds a servomotor to the 3-cable drive, and an extra cable is needed in the soft arm, which takes up more space.

The traditional modeling methods of the soft arm include analytical modeling using the physical parameters of the soft arm. The most used method is the piecewise constant curvature method proposed by Ian Walker. This method is simple and practical and has a wide range of applications (Lafmejani et al., 2020). At the same time, to improve the accuracy of modeling, many scholars have also proposed more complex modeling methods, such as the variable curvature method and the Cosserat rod method. A unified Cosserat-based formulation derived by resorting to a coupled approach that comprises of a model of the structural dynamics of the cephalopod-like elastic bladder and a model of the pulsed-jet thrust production is presented and tested by the robotics artefact developed by the authors sucessfully (Renda et al., 2015; Renda et al., 2018). A novel generation of macroscale underwater propellers is designed, and a Cosserat-based model is presented, accurately describing, and predicting the kinematic and the propulsive capabilities of the proposed solution (Armanini et al., 2021). However, due to the large amount of calculation and the number of parameters that need to be identified, these methods have not obtained significant performance improvement, so they are not widely used (Kim et al., 2021). Traditional modeling methods are difficult to build accurate models due to internal nonlinear interference and lack robustness and portability between different prototypes. Therefore, researchers turned their attention to machine learning, trying to use machine learning methods to build and control the soft arm model. As we all know, machine learning algorithms will effectively solve nonlinear problems in various fields. The neural network is first used in learning the forward kinematic model to solve the control problem of the cable-driven soft arm (Giorelli et al., 2013). A model-free control method based on reinforcement learning is proposed and this method is implemented on a multi-segment soft arm on a two-dimensional plane (You et al., 2017; Jiang et al., 2020). The prototype experiment verified the effectiveness and robustness of the control strategy and designed a simulation method to accelerate the training process. An octopus-inspired robot combines swimming and 4-leg crawling locomotion is designed and a Least Squares-based method coupled with a Genetic Algorithm-based method is employed for two phases, respectively (Giorgio-Serchi, et al., 2017). A systematic method for soft robot underwater locomotion using a controller based on deep reinforcement learning as a framework is developed and verified to create control inputs. However, it still didn’t expand the working space to three dimensions (Li et al., 2021).

In this paper, combined with the appearance and movement characteristics of the octopus’s tentacle, a more streamlined 3-cable cable-driven soft arm is designed and fabricated, and the soft arm model is established through a data-driven modeling method. At the same time, the control method of deep reinforcement learning is extended to three-dimensional space, to realize the straight walking, left turn and right turn of the biped robot.

## Design and Fabrication of the Soft Arm

**FIGURE 1:**
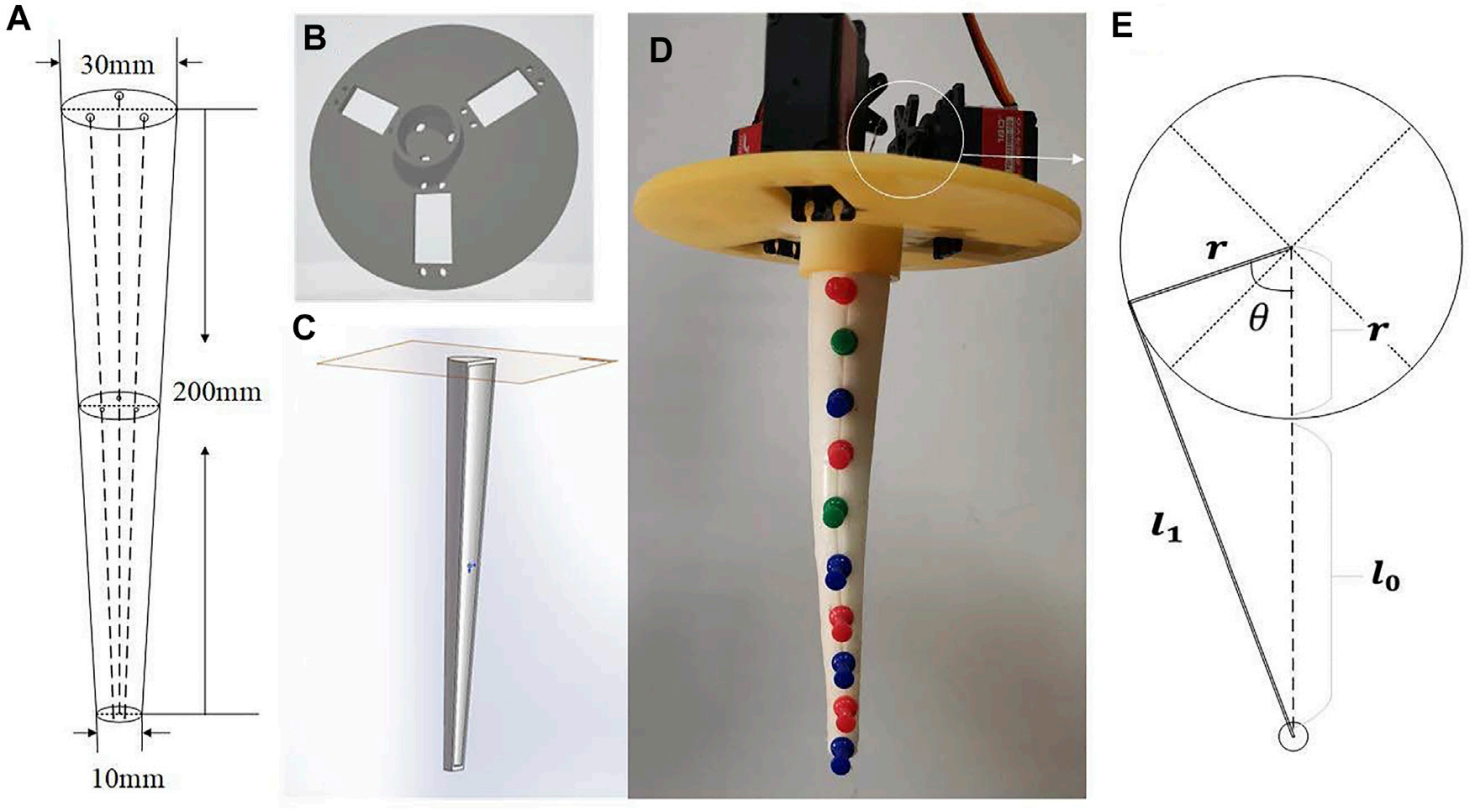
Design and fabrication of the soft arm. **(A)** Structure design of a cable-driven soft arm **(B)** The mold of the soft arm **(C)** The mold for bracket **(D)** The assembly of the soft arm **(E)** The link relationship of the driving cable.

Imitating the movement mechanism and structural characteristics of the octopus tentacles, a soft arm was designed as Figure 1A. Its distal radius is 15 mm, proximal radius is 5 mm and length is 200 mm. The taper angle of the soft arm is 5.71°. It can achieve a bending situation similar to an octopus tentacle, with a small degree of bending at the proximal end and a large degree of bending at the distal end.

The mold of soft arm is designed as Figure 1B. The inner diameter of the two ends and length of the mold are the dimensions of the soft arm mentioned above. Lay the cables respectively in two 3D-printed molds, pour silica gel, demold after curing and assemble. As is shown in Figures 1A,C platform to fix servomotors and soft arm is designed, and three servomotors are symmetrically distributed at 120° with respect to the center. The assembled soft arm is shown in Figure 1D, and ten marking points are marked equidistantly on the central axis.

Three driving cables are respectively controlled by three waterproof servomotors. By setting different pulse width x of the input signal of the servomotor, it can be controlled to achieve different rotation angles. The type of the servomotor is JX6621, the pulse period is 20 ms, and its rotation range is 180°. The corresponding relationship between the servomotor parameter x ( 0.5ms−2.5ms ) and rotation angle of the servomotor θ ( 0−180° ) is as follows:

$$\theta =-\frac{\pi }{2}x+\frac{5\pi }{4}.$$ (1)

According to Figure 1E, the relationship between the pulling length of the cable ΔL and the rotation angle θ of the servomotor can be obtained through the law of cosine:

$$\Delta L=l_{1}-l_{0},$$ (2)

$$l_{1}=\sqrt{r^{2}+{\left(r+l_{0}\right)}^{2}-2r\left(r+l_{0}\right)\mathrm{cos\theta}},$$ (3)

where r=16mm is the radius of the turntable driven by the servomotor, $l_{0}=15mm$ is the distance from the fixed place of the servomotor of the cable to the hole of the distal end of the corresponding soft arm for cable when not pulling, and $l_{1}$ is that distance when pulling the cable.

**FIGURE 2:**
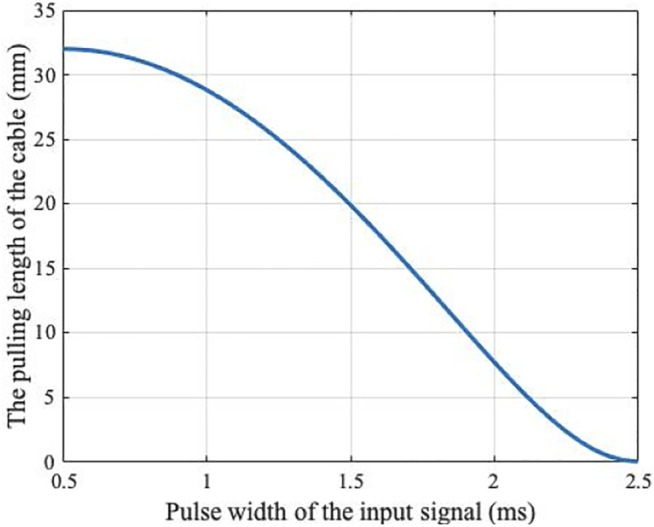
The relationship between the parameter of the servomotor and the pulling length of the cable.

Combining the above three formulas, the parameters of the servomotor x can be converted into the pulling length of the cable ΔL (Figure 2). When the parameter of the servomotor changes from 2.5ms to 0.5ms , the pulling length of the cable ranges from 0mm to 32mm .

## Modeling and Control of the Soft Arm

### Modeling of the Single Soft Arm Based on Data-Driven Model

**FIGURE 3:**
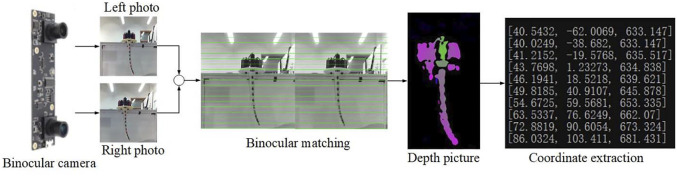
Image processing of the posture of the soft arm.

The most important part of training the neural network is collecting valid data. In this paper, we collect the data in the real-world by using camera. The advantage of this method is that it is easier to acquire high-quality position data ( px,py,pz) of the points in the soft arm. Aiming at the three-dimensional modeling requirements of the soft arm, ordinary monocular cameras can only obtain two-dimensional image information, which cannot effectively meet the needs of obtaining position depth. Therefore, a binocular camera is used. The data-collecting system hardware consisted of a binocular camera, a calibration board, a notebook computer, and a supporting structure. After the calibration is completed, use the binocular camera to shoot the soft arm, and then use the SGBM algorithm to perform binocular matching to generate a depth map. At this time, the three-dimensional coordinates of the marker points can be extracted as Figure 3.

A data-driven model based on the Keras library of Python is built. Because there are three cables, the parameters of the servomotor 1, the servomotor 2 and the servomotor 3 are set as the input x1,x2,x3 of the designed neural network. The hyper-redundancy of the octopus and the lack of limitation by the number of skeletal joints make the representation of information in body coordinates unrealistic (Nesher et al., 2020). Another method is needed to represent the posture information of the soft arm. Because the soft arm does not have clear joints and the body deforms continuously during moving, the movement of a point on the soft arm at a certain time and the movement in a short interval near that point can be regarded as approximately the same. Therefore, a series of discrete points are selected as the control object on the soft manipulator. The experimental results also prove that this selection can meet the purpose of modeling. To facilitate the calculation, when seeking the constant curvature kinematics model, the manipulator is divided into n constant curvature sections, and the feedback point is set at the cross section of each constant curvature section (Ni et al., 2017). Therefore, pushpins are used to mark five feature points on the vertical center line of the soft arm at equal distances to feedback the degree of bending. Based on the above preprocessing, the output layer of the neural network is composed of 15 neurons ( px1,py1,pz1,…,px5,py5,pz5 ), which in turn are the three-dimensional coordinates of five feature points.

**FIGURE 4:**
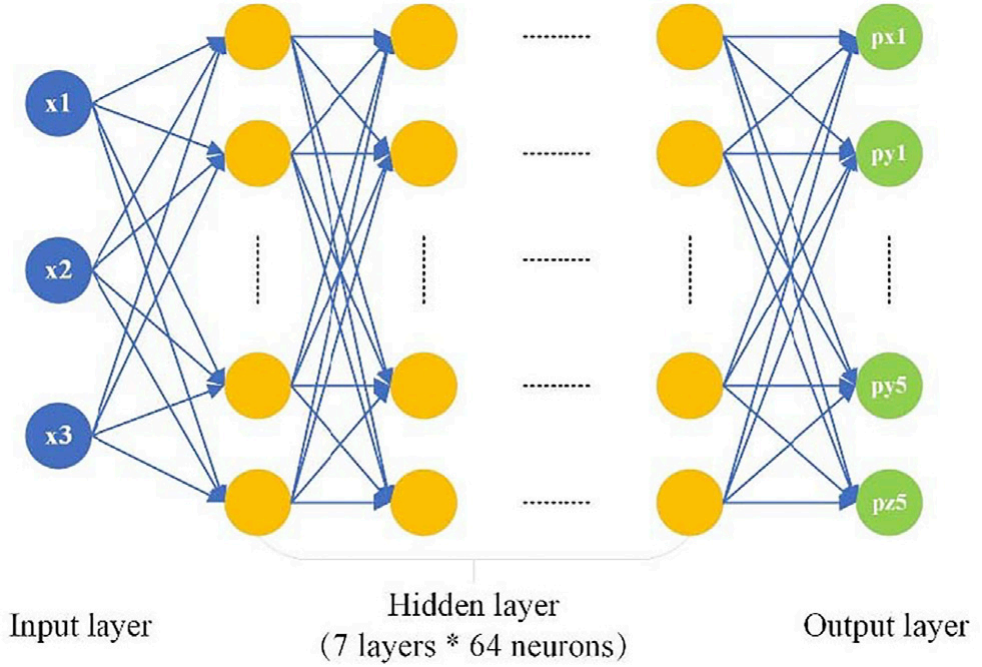
Structure design of the neural network of the soft arm.

To enable the network to fully learn the model features, gradually increase the number of hidden layers and the number of neurons. The accuracy of the network reaches a performance bottleneck when there are seven layers and 64 neurons in each layer, and then it tends to be saturated. Therefore, the hidden layer of the neural network is designed (Figure 4).

In this paper, Tanh function is selected as the activation function, the mean square error function is used as the training loss function, and Adam is used as the optimizer to train the above-mentioned deep neural network.

First, control the servomotor to traverse the reachable space of the entire soft arm with the same interval, and use the binocular camera to capture the bending shape of the soft arm each time, and save the soft arm shape corresponding to each action. Then, extract the morphological characteristics of the soft arm, and the available image data are screened. Finally, the pulling length of the cables are used as input, and the morphological feature data of the soft arm are used as the output to train the data-driven model of the soft arm, and the simulation environment of the soft arm is obtained. When training the neural network through the random gradient descent method (SGD), a data set of images of the soft arm is constructed, and 85% of images are randomly selected as the train set of the neural network, and 15% of images are used as the test set. This method solves the problem of difficult modeling of the soft arm and establishes the multi-layer perceptron model of the soft arm through the data collected in the experiment.

**FIGURE 5:**
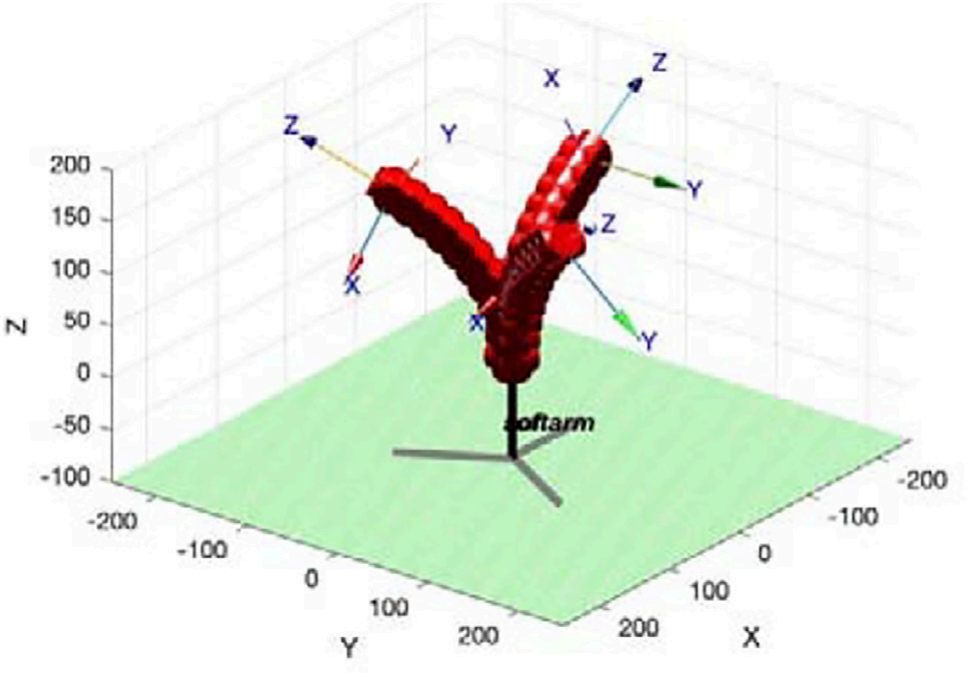
Simplified description of the data set.

To reduce the data dimension and speed up the training speed, the data set here refers to the pose information of the soft arm when a certain driving cable is pulled alone to exhibit different degrees of bending. As shown in Figure 5, one driving cable is pulled individually to make it bend 120° to each other to form a motion primitive.

**FIGURE 6:**
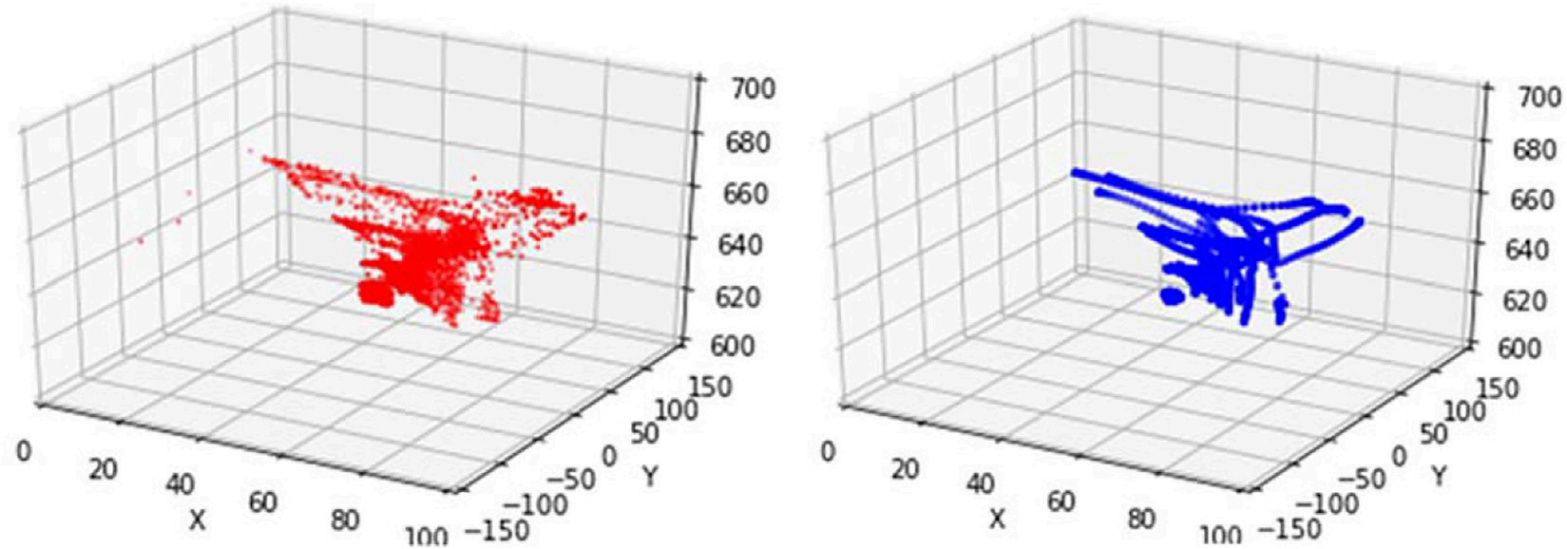
Comparison of the trajectory of the soft arm before and after training.

As shown in Figure 6, the trajectory map (red) before training is slightly disorganized, and it cannot be clearly seen that the bending directions are 120° from each other when pulling one cable. The trajectory map (blue) after training effectively eliminates abnormal pose information, and more intuitively shows the movement trajectory characteristics of the soft arm under the 3-cable drive.

**FIGURE 7:**
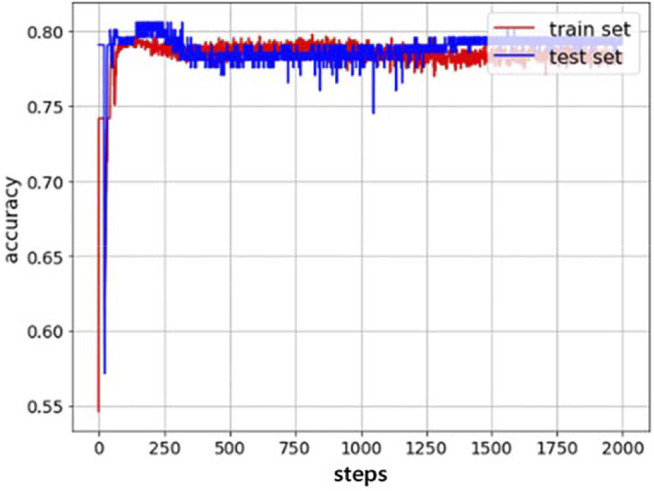
Accuracy of train set and test set of the trajectory of the soft arm.

As is shown in Figure 7, the experimental results of the space soft arm (that is, the soft arm in the air) verify the validity of the established inverse kinematics model and the rationality of the soft arm positioning control method based on this model.

We also accomplish the same experiments under the water for the movement of the space soft arm and the underwater soft arm, we pulled the drive cable with the same step to complete two sets of experiments. The experimental results show that the data-driven model is also suitable for the underwater movement of the soft arm, but the time that underwater soft arm completes the bending action is longer than the time for the space soft arm to complete the action. When the same servomotor control parameters are executed at the same time, the static bending degree of the underwater soft arm after the completion of the action is greater than the static bending degree of the space soft arm. When the drive cable is pulled with the same step, the data distribution is dense at both ends and sparse in the middle, which is synchronized with the change in the pulling length of the cable.

### The Single-Arm Control of Three-Dimensional Deep Reinforcement Learning

The idea of reinforcement learning (RL) comes from zoology theory and conditioning theory. It is a kind of bionic algorithm that people get through the study of animal learning. RL relies on exploratory learning to give robots the ability to learn adaptively and can solve the problems of complex design process, and lack of robustness and autonomy in traditional control algorithms.

In this paper, Deep Q-Network (DQN) in RL is used as the soft arm control algorithm. DQN is the combination of Q-Learning and neural network, turning the Q table of Q-Learning into Q-Network. The use of deep neural network to approximate the Q table enables Q-Learning not only to process continuous state spaces, but also to have a certain generalization ability, which effectively enhances the application range of traditional Q-Learning (Zhang et al., 2015). Q-learning is a dynamic programming method based on value iteration. The function follows the following update formula:

$$Q_{t}\left(s_{t},a_{t}\right)\leftarrow Q_{t}\left(s_{t},a_{t}\right)+\alpha \left(r_{t}+\mathrm{\gamma ma}x_{a}Q_{t+1}\left(s_{t+1},a\right)-Q_{t}\left(s_{t},a_{t}\right)\right),$$ (4)

where $Q_{t}\left(s_{t},a_{t}\right)$ is the Q function, r is the reward, α is the learning rate, and γ is the attenuation coefficient.

In this paper, the parameters of training are shown in Supplementary Table S2. The posture control of the soft arm is a process of continuous exploration. To improve the training effect of deep reinforcement learning, it is necessary to design an appropriate reward function (Supplementary Table S3) to adjust the control strategy and an end sign of the current training around. When the error of the posture of the soft arm is smaller than the set threshold, or the number of steps exceeds the set maximum running times, this round will be stopped.

**FIGURE 8:**
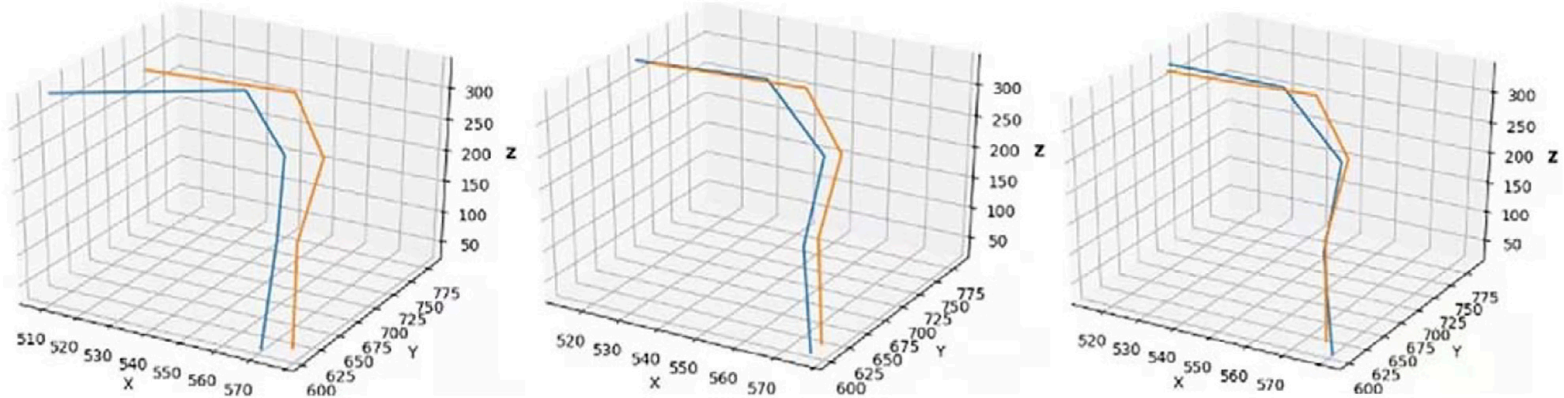
The training process of the soft arm in a certain round.

The DQN training algorithm proposed is based on the greedy strategy. When training the neural network each round, a set of initial poses and a set of end poses are randomly selected from the data set. At the beginning, random actions are selected with a greater probability to enhance the DQN to explore the surroundings. Late in training, the optimal control action is selected with a greater probability. It is helpful to jump out of the local optimum and find the global optimum. The training process of a certain round is as follows, the line connected by orange marking points is the target posture of the soft arm model, and the line connected by blue marking points is the current posture of the soft arm model. The current posture keeps getting closer to the target posture through training, as shown in Figure 8.

**FIGURE 9:**
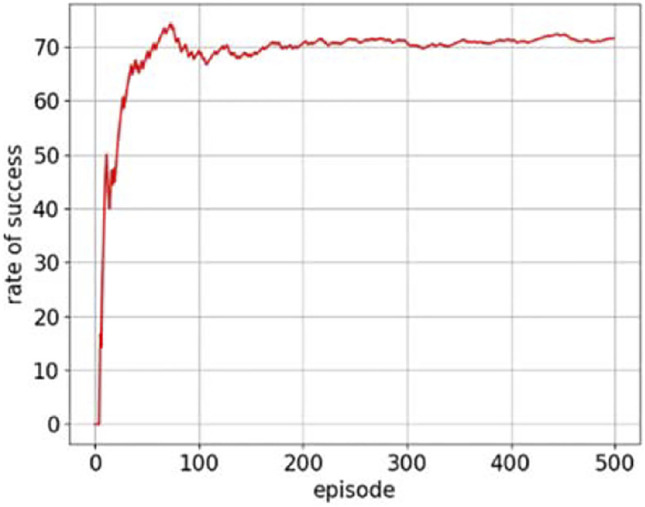
The rate of success in the training process of the soft arm.

The rate of success in the simulation training process is shown in Figure 9. As the number of learning increases, the rate of success fluctuates and eventually stabilizes at 71%. Therefore, the effectiveness of this DQN-based reinforcement learning method in controlling the posture of the soft arm has been verified in the simulation environment.

## Motion Coordination and Gait Design of a Bipedal Walking Soft Robot Bio-Inspired by Octopus

### The Design of the Octopus-Inspired Soft Robot

Among different locomotion, friction-based gaits are among the slowest forms of locomotion employed, whereas running, jumping, and flying are among the fastest. Walking and swimming are intermediate between these border categories (Calisti et al., 2017). An underwater legged locomotion by means of a robotic octopus-inspired prototype and its associated model was studied (Calisti et al., 2015). And the mass of the robot is 0.755 kg and the length of the legs is 0.3 m. Finally, the robot can achieve an average speed of 4.2 cm/s.

**FIGURE 10:**
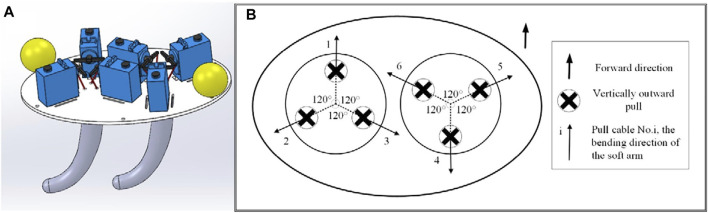
**(A)** The assembly of the octopus-inspired soft robot **(B)** Distribution of double-arm drive cables, tension direction and bending direction.

In this paper, a biped walking (or running) soft robot is designed as Figure 10A. The height of the robot is 0.2 m and the mass of that is 0.4 kg. The length of the legs is 0.15 m and the taper angle of that is 7.63°. The taper angle of the soft arm that composes the soft robot is larger than the single soft arm in the previous experiment to support the robot platform better. And two same balloons are equipped in a symmetrical position on the robot platform to make the robot balance. When conducting the robot underwater bipedal walking experiment, the robot needs to be balanced in the vertical direction. In the vertical direction, the robot is subject to its own gravity and buoyancy by these balloons with a radius of 3.5 cm.

**FIGURE 11:**
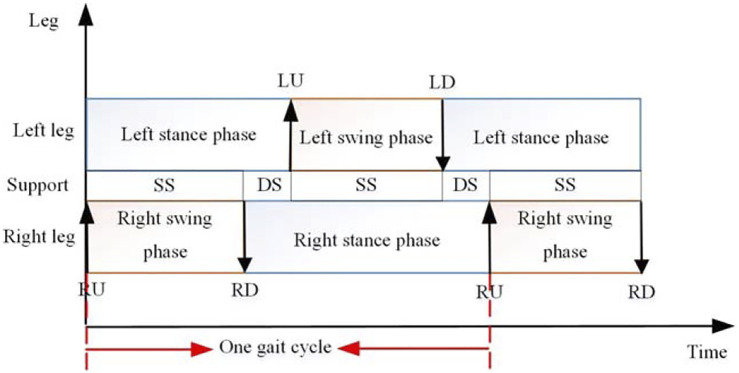
Bipedal locomotion stride for *Amphioctopus marginatus* Octopus

The bipedal walking method of an octopus is different from the common crawling method. The bipedal walking process is shown in Figure 11. RU means lifting the right leg, RD means putting down the right leg, LU means lifting the left leg, LD means putting down the left leg, SS means single support, and DS means double support (Wu et al., 2021).

Inspired by the biped walking gait of Amphioctopus marginatus Octopus (Figure 11), the walking gait of the biped robot is planned. The walking gait relies on the effective control of the six drive cables in the two arms. The distribution position of these cables is shown in Figure 10B. Three drive cables labeled 1, 2 and 3 control one arm, and the other three drive cables labeled 4, 5 and 6 control the other arm. The pulling direction is all perpendicular to the outside, and the arrow is the bending direction of the soft arm when the corresponding drive cable is pulled. At the same time, the bending direction of the No. 1 drive cable is designated as the front side, that is, the forward direction.

**FIGURE 12:**
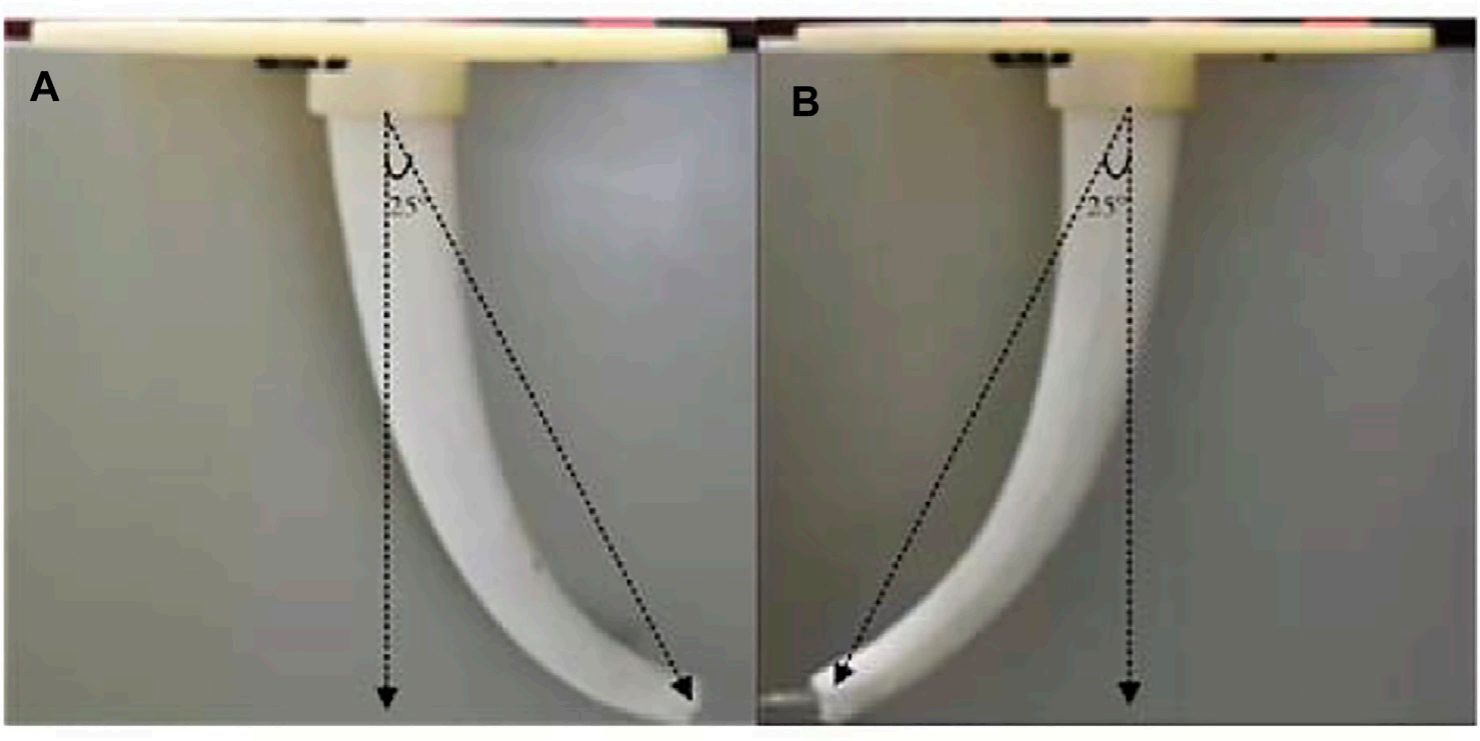
Bending comparison of single arm. **(A)** Pull the No. 1 drive cable by 32 mm **(B)** Pull the No. 2 and 3 drive cables by 32 mm respectively.

Taking a single arm as an example (Figure 12), under the same parameter conditions, the effect of pulling No. 2 and No. 3 at the same time is the same as the effect of pulling No. 1 only. When the drive cable is pulled 32mm, the bending angle is 25°, and the direction difference is 180°.

In the same way, for biped robot, the effect of pulling No. 5 and 6 at the same time is the same as the effect of pulling No. 1 only, and the direction is also the same.

### The Gait Design of Straight Walking of the Octopus-Inspired Soft Robot

The original state is that the robot’s legs are slightly bent forward, and both legs are in contact with the bottom of the water tank.

Three individual actions of single arm are ordered as following.

Action 1: Stretch No.1 drive cable and loose No.2 and No.3 drive cables to make one soft arm bend forward. Action 2: Loose No.1 drive cable and keep No.2 and No.3 drive cables as last action to make the soft arm contact with the ground. Action 3: Stretch No.2 and No.3 drive cables and loose No.1 drive cable to make one soft arm bend back.

According to the label of the six cables in Figure 10B, the motion cycle of the robot’s straight walking is ordered into four sequences as following.

Action 1: The soft arm controlled by No.1∼3 drive cables (the No.1 soft arm) bends forward and leaves the ground, and the soft arm controlled by No.4∼6 drive cables (the No.2 soft arm) relaxes and keeps in contact with the ground. Action 2: The No.1 soft arm keeps the previous stage of bending, and the No.2 soft arm bends backward and pushes the ground to generate and forward thrust, which pushes the soft robot forward by half a step. Action 3: The No.1 soft arm relaxes and returned to contact with the ground, and the No.2 soft arm bends forward and leaves the ground. Action 4: The No.1 soft arm bends backward and pushes the ground to generate forward thrust, which pushes the robot forward to complete a step, and the No.2 soft arm keep the state of the previous stage.

When the robot goes forward, the drive status of six cables is shown in Supplementary Table S4 briefly to implement the above actions. “F” represents stretching the cable to make one soft arm bend forward, “B” represents stretching the cable to make one soft arm bend back, and “O” represents the original state or last state.

**FIGURE 13:**
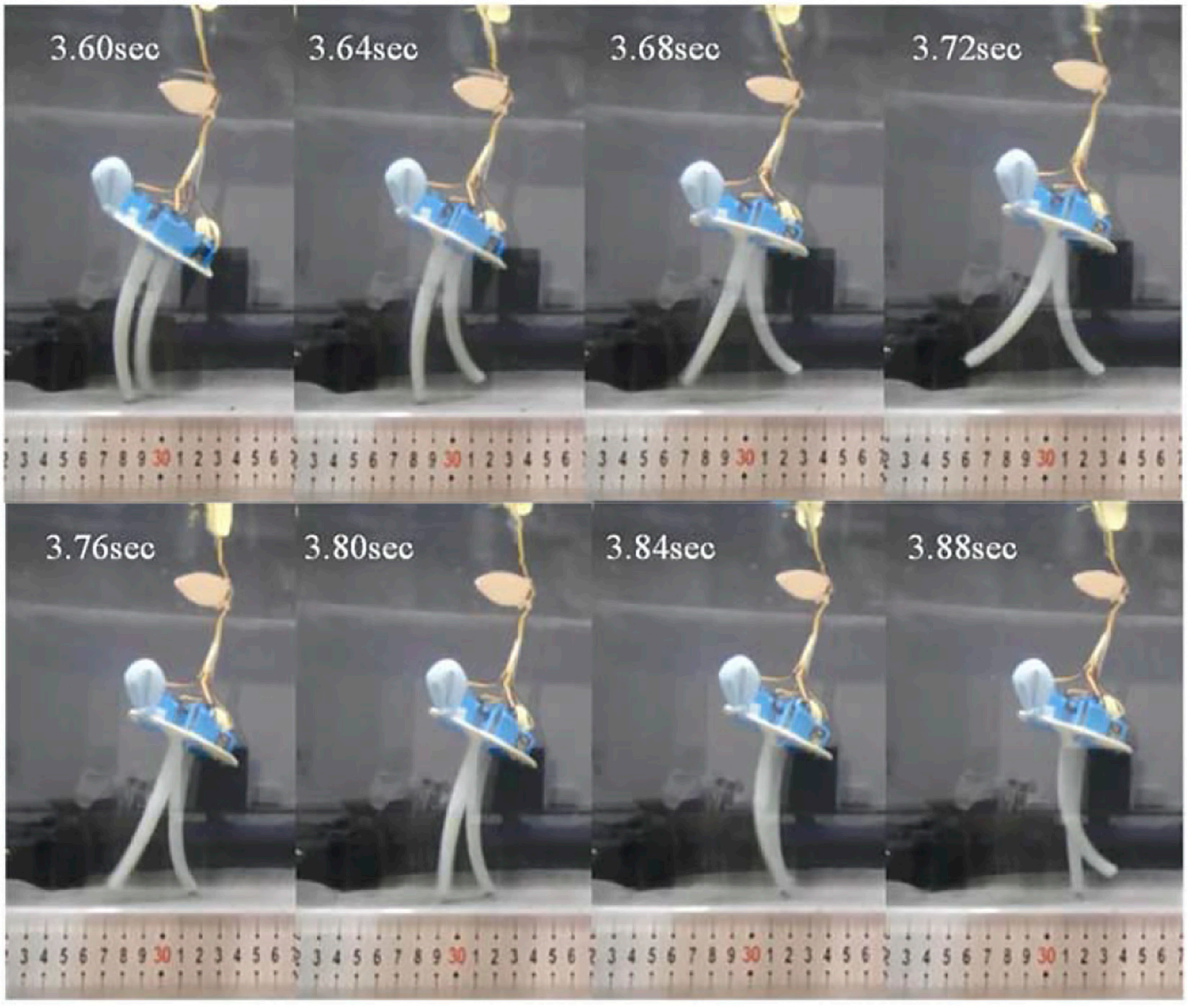
Frame-by-frame analysis of the video of the straight walking on the flat ground.

The robot was placed in a water tank with a length of 80 cm, a width of 45 cm, and a depth of 45 cm, so that its body was completely submerged in water, and its soft arms were kept in contact with the flat ground under the water. According to the 6-cable control commands corresponding to the robot’s straight walking gait planned above, the underwater bipedal walking experiment was carried out (Figure 13).

**FIGURE 14:**
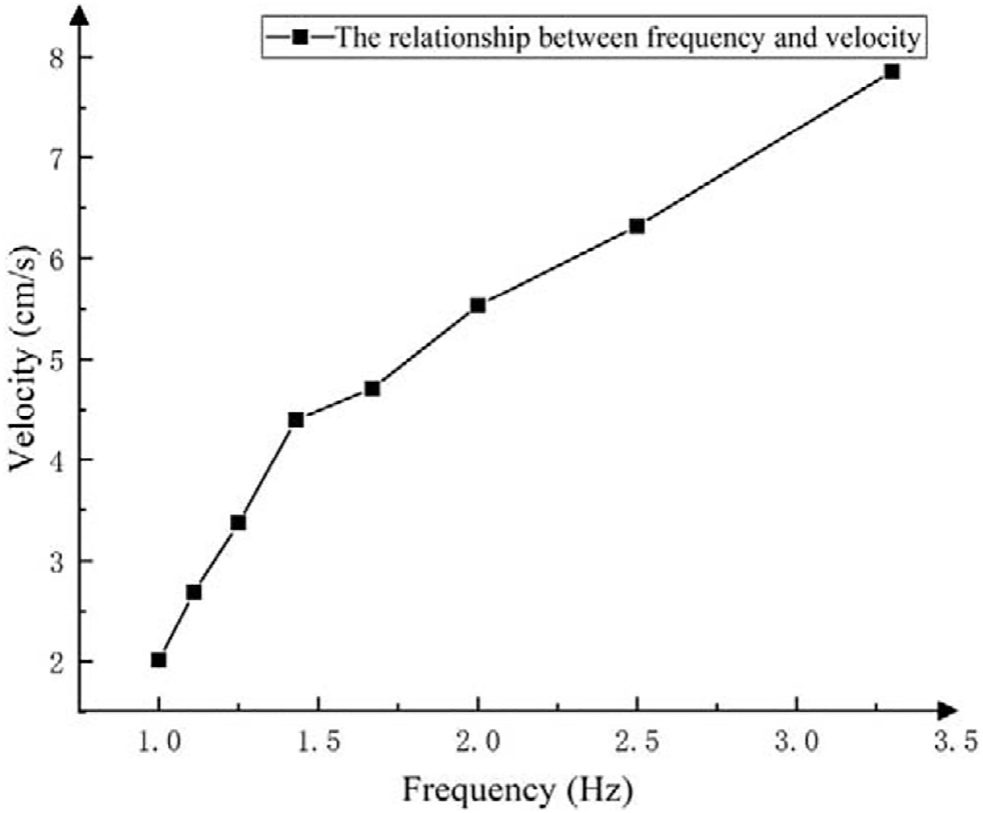
The relationship between motion velocity and the frequency.

Using the 6-cable driving control commands corresponding to the straight walking gait of the robot planned in Supplementary Table S4, adjust the motion period every 0.1 s between 0.3 and 1 s, and complete ten walking experiments at each period. Record the time and distance in turn to calculate the walking speed of the robot under the corresponding motion period. And then, turn the motion period into motion frequency to express it more formally. According to the law of 3σ , the average speed after excluding the abnormal value is obtained as the average walking speed at different motion frequency (Supplementary Table S5). As is shown in Figure 14, the motion velocity of the biped robot is approximately positively proportional to the frequency.

**FIGURE 15:**
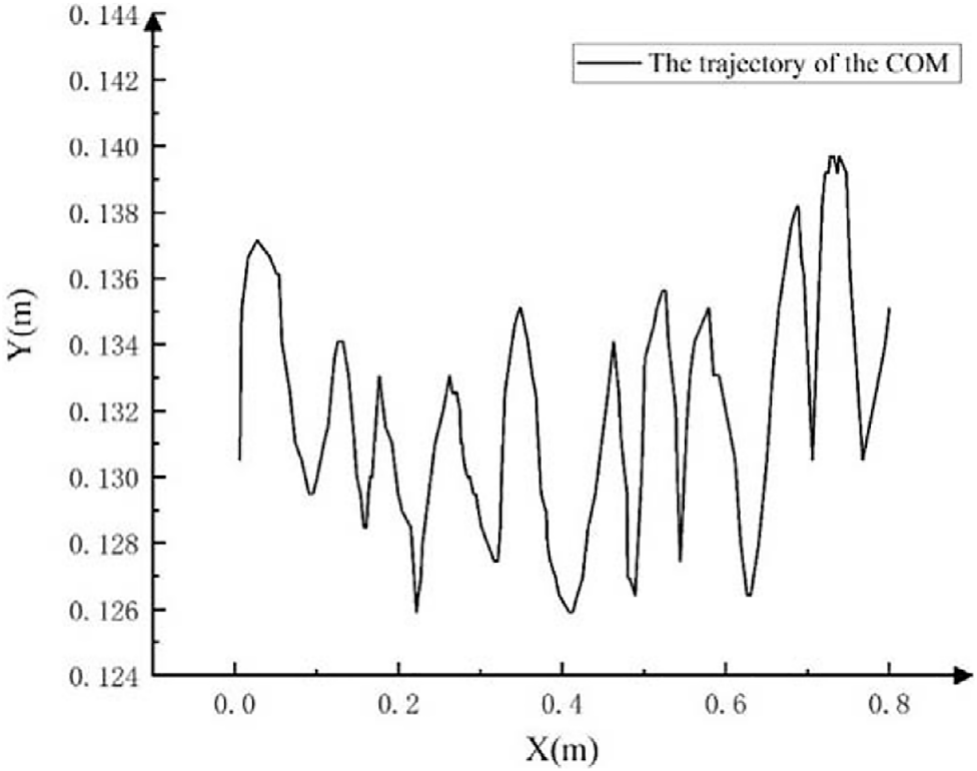
The straight walking trajectory of the center of mass of the robot on the flat ground.

Based on the image information captured by the camera, the machine vision algorithm is used to extract the straight walking trajectory of the center of mass of the robot in an underwater flat environment, as shown in Figure 15. The forward direction of the robot is the positive direction of the *X*-axis. Overall, the robot has a good straight walking gait, and the movement is relatively stable. In the water tank environment with a length of 0.8 m, the center of mass of the robot has experienced a total of 11 up-and-down motion links during the straight-line walking process, that is, 5.5 motion cycles. Among them, the average peak-to-peak value of the robot’s center of mass fluctuation is 0.65 cm, and the average forward distance of each motion cycle is 14.5 cm.

**FIGURE 16:**
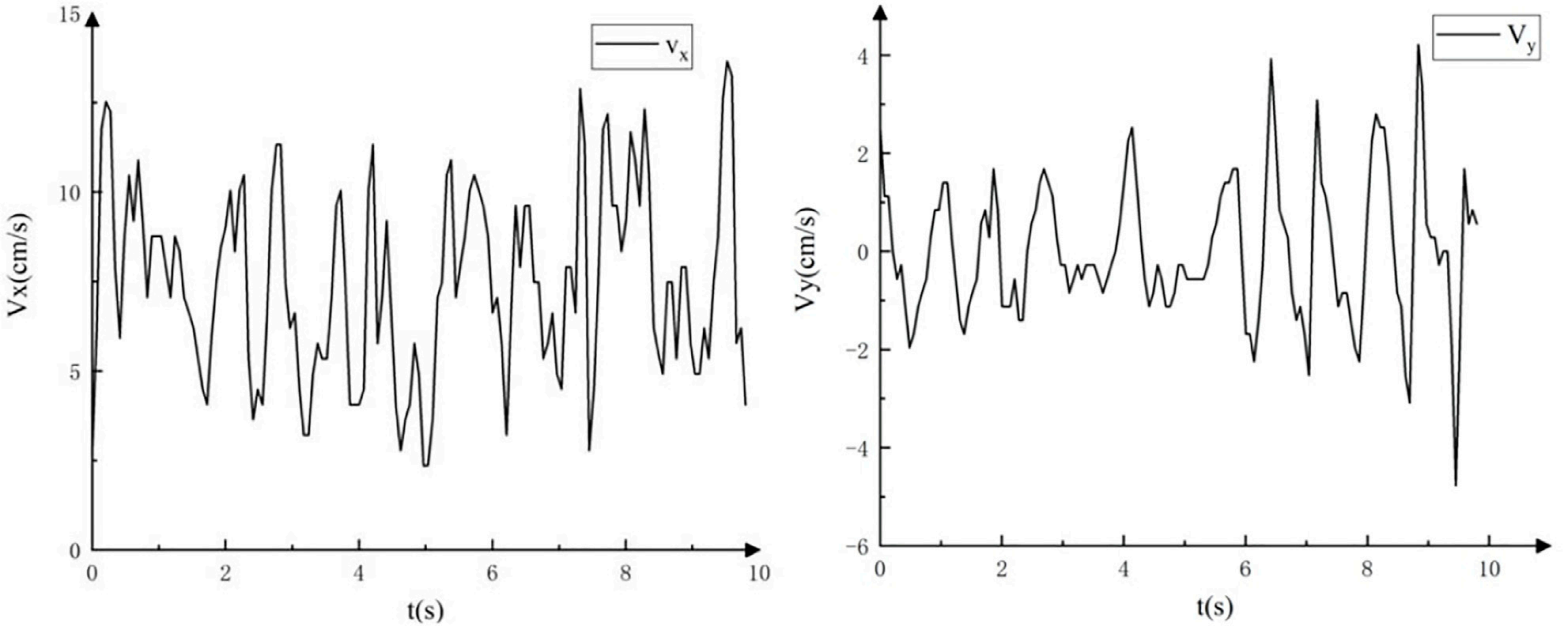
The velocity of the robot in horizontal and vertical directions when straight walking on the underwater flat ground.

The velocity changes in the horizontal and vertical directions when straight walking on the underwater flat ground. The movement speed fluctuates regularly in Figure 16.

In each motion cycle, the maximum instantaneous speed of the robot in the *x*-axis is generated at the highest point of the motion trajectory. The robot can achieve an average speed of 7.78 cm/s, and the maximum instantaneous speed can reach 12.8 cm/s.

**FIGURE 17:**
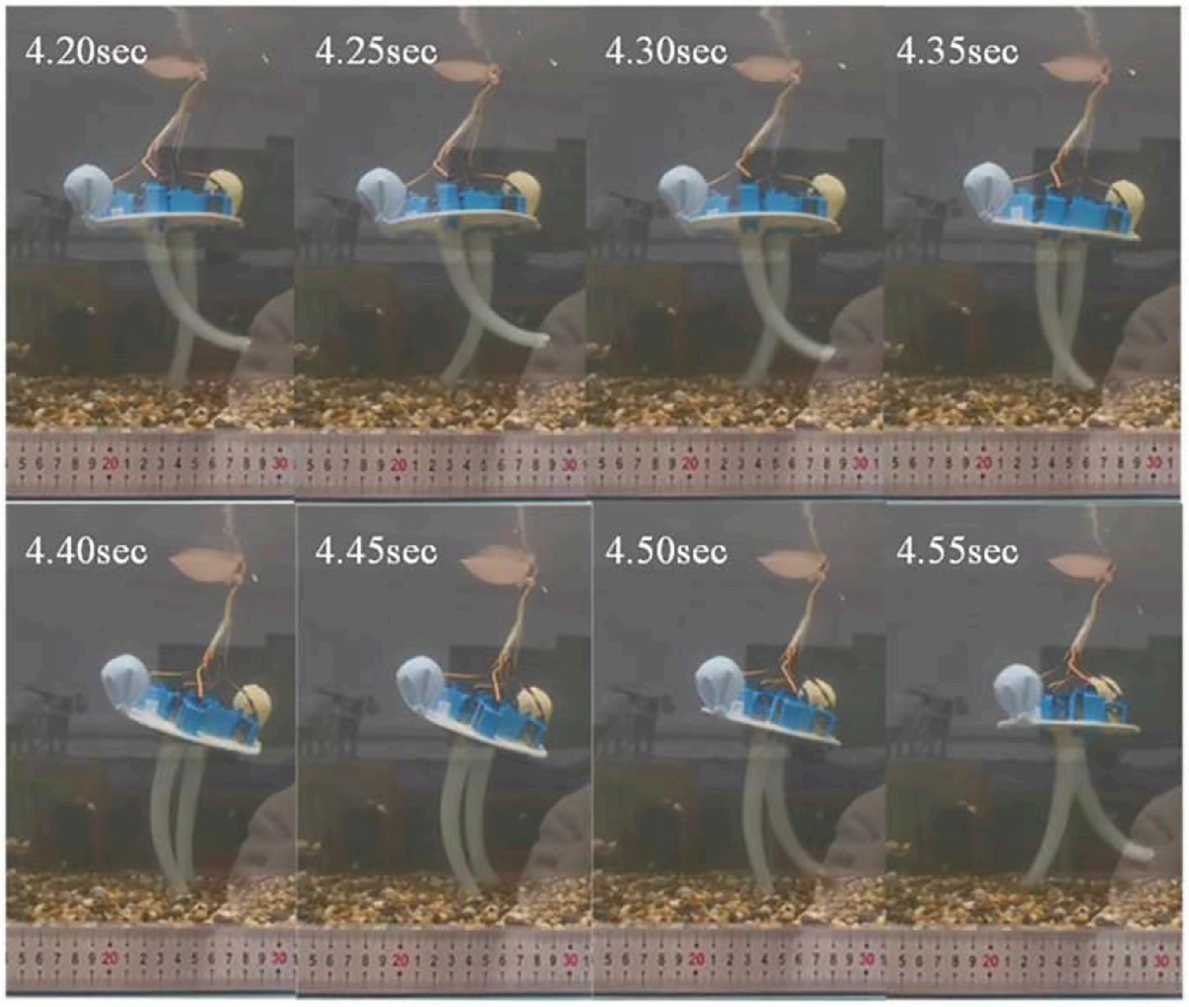
Frame-by-frame analysis of the video of the straight walking on the sandy ground.

To test the locomotion ability of the bionic octopus biped walking robot in a complex environment, and to reflect the robustness of its walking action underwater, and to be closer to the real seabed environment, a thickness of 1∼2 cm sand was laid at the bottom of the original water tank. According to the 6-cable drive control commands corresponding to the straight walking gait of the robot planned in the previous section, and the motion period of a single soft arm of the robot is set to 0.3 s, the underwater bipedal walking experiment is carried out in a sandy underwater environment. Figure 17 shows the running state of one motion period of the robot. After many experiments, the average speed of the robot in the underwater sand environment is 5.3 cm/s.

**FIGURE 18:**
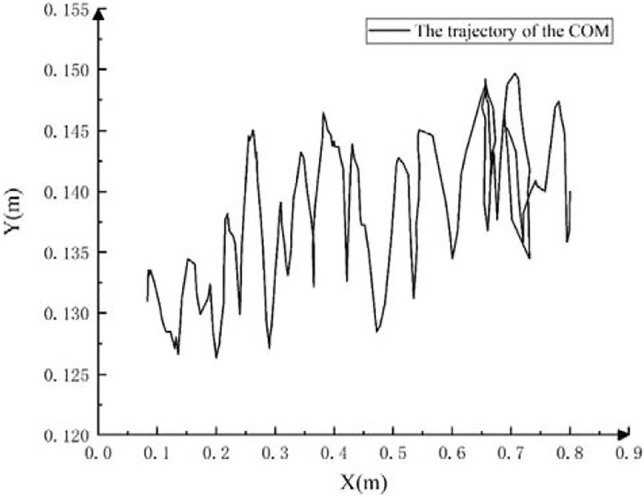
The straight walking trajectory of the center of mass of the robot on the sandy ground.

According to the captured motion image information of the robot, the straight walking trajectory of the center of mass of the robot in the underwater sandy environment is extracted, as shown in Figure 18. Although the sandy environment has a certain impact on the robot’s bipedal straight walking, in general, the fluctuation of the motion state is still relatively stable. In the underwater sandy environment with a length of 0.8m, the center of mass of the robot has experienced a total of 16 up-and-down motion during the straight walking process, that is, eight motion cycles. Affected by the uneven height of the sandy ground, the average peak-to-peak value of the robot’s center of mass fluctuation increases to 0.95 cm. Affected by the resistance of the sandy ground, the average forward distance of the robot in each motion period is reduced to 10 cm compared to the flat environment. Despite some resistance and slippage, the motion of the robot is overall stable in the sandy ground. There is a fluctuation of the motion trajectory of the robot because of the sand laying problem.

## Summary and Outlook

According to the motion characteristics of the octopus’s tentacle, a data-driven model between its parameter control and the three-dimensional posture of the soft arm is established based on the TensorFlow framework. And DQN strategy in deep reinforcement learning is used to train the model to control the actual posture of the soft arm. This modeling and control method are used in the octopus-inspired biped robot, and the walking gait of the robot is designed. By observing and analyzing multiple experiments of underwater biped walking experiments in the water tank, the rationality of the gait design of the robot is confirmed. The average speed of the bipedal octopus walking robot can achieve an average speed of 7.86 cm/s when straight walking, and the maximum instantaneous speed can reach 8.5 cm/s. At the same time, it can also be fast and stable when turning around.

Compared with other underwater robot, the main advantages of the robot in this paper are as follows:

1) The crawling mechanism, manipulating arm and swimming mechanism of the POSEIDRONE robot (Calisti et al., 2015) are independent and decoupled from each other, which reduces the difficulty, but the complexity is high.
2) What’s more, another bipedal walking robot (Portilla et al., 2019) is hydraulicly driven, and the SLIP model of land bipedal walking is extended and applied to underwater bipedal walking control.
3) The structure of SILVER and SILVER2 robots is a kind of legged rigid structure which only have two degrees of freedom.

Compared with the above three kinds of legged robots, this paper adopts the bionic octopus flexible arm to operate and bipedal walking respectively. Compared with the rigid structure, it fits more closely with the environment, has less impact on the environment, and is more friendly.

In the future, cameras and IMU modules could be added to the platform to realize the underwater target recognition and autonomous navigation functions of the bionic octopus robot. Then, it could be applied in more work scenarios and achieve greater value. What’s more, the robustness to impact disturbances of the robot is needed to improve. We will learn from that a new neural network enhanced control system that stabilizes a three-dimensional simulated biped model of a human wearing an exoskeleton is presented (Liu et al., 2021). Results show that it stabilizes human/exoskeleton models and is robust to impact disturbances.

## Data Availability

The original contributions presented in the study are included in the article/Supplementary Material, further inquiries can be directed to the corresponding author.
