# Supplementary figures and images for: Bipedal Walking of Underwater Soft Robot Based on Data-Driven Model Inspired by Octopus
Source: Front Robot AI. 2022 Apr 20;9:815435. doi: 10.3389/frobt.2022.815435 (PMC9065362; doi:10.3389/frobt.2022.815435)

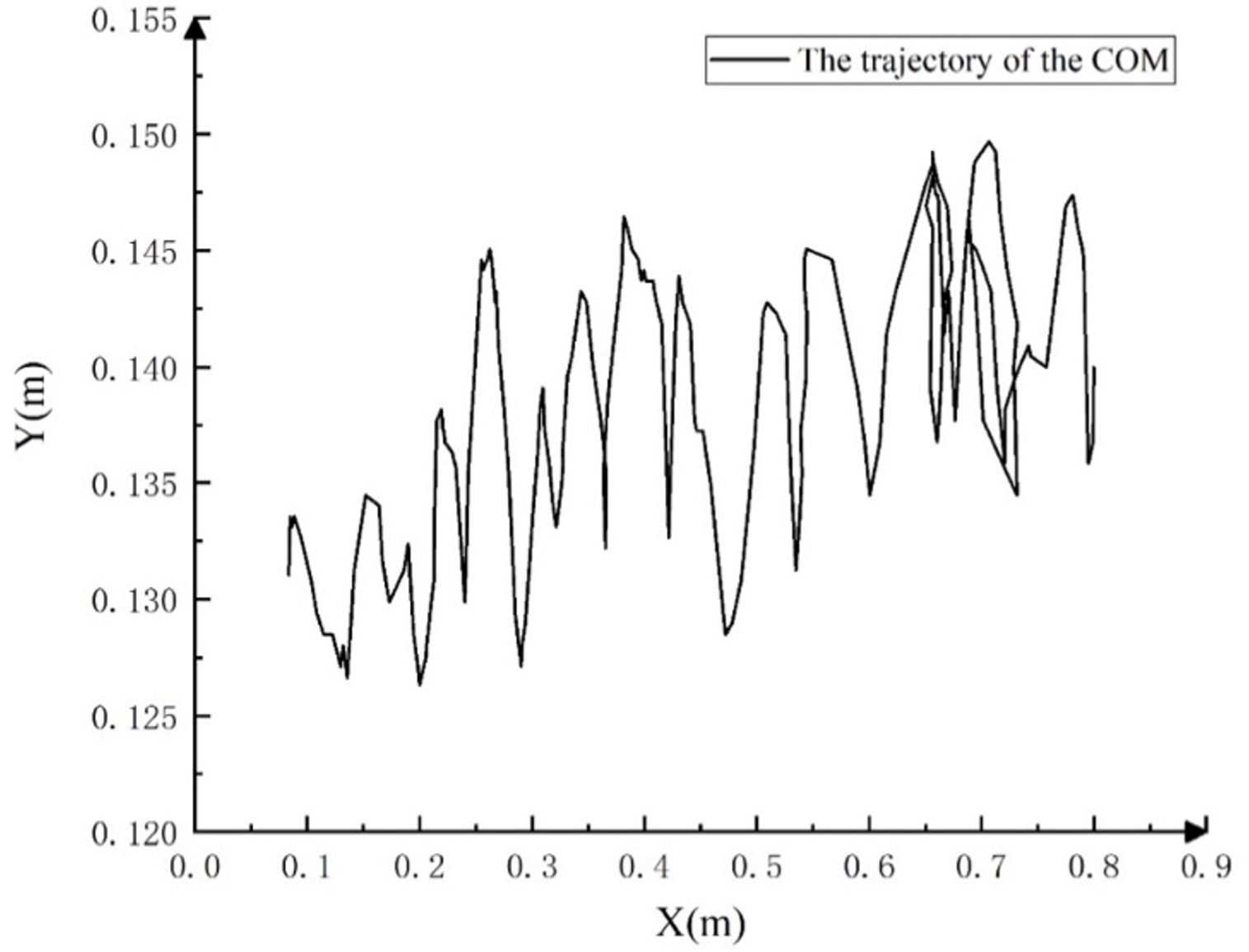

Supplement: Supplementary file 1 [file Image3.jpg]

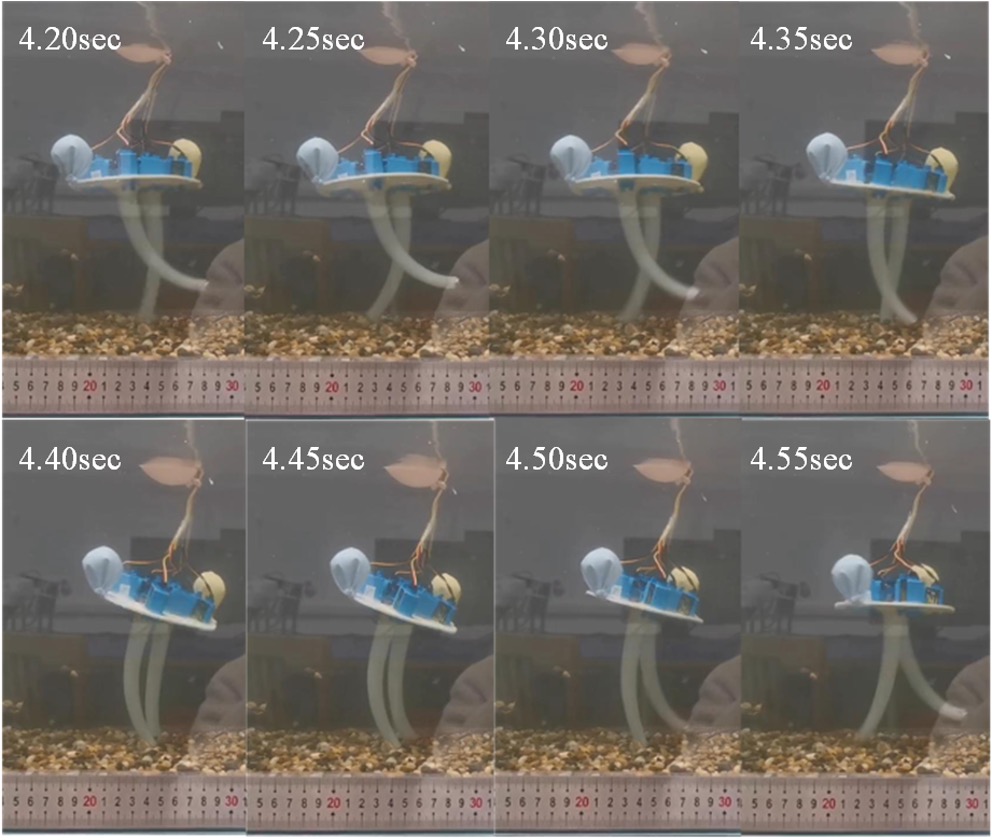

Supplement: Supplementary file 2 [file Image2.jpg]

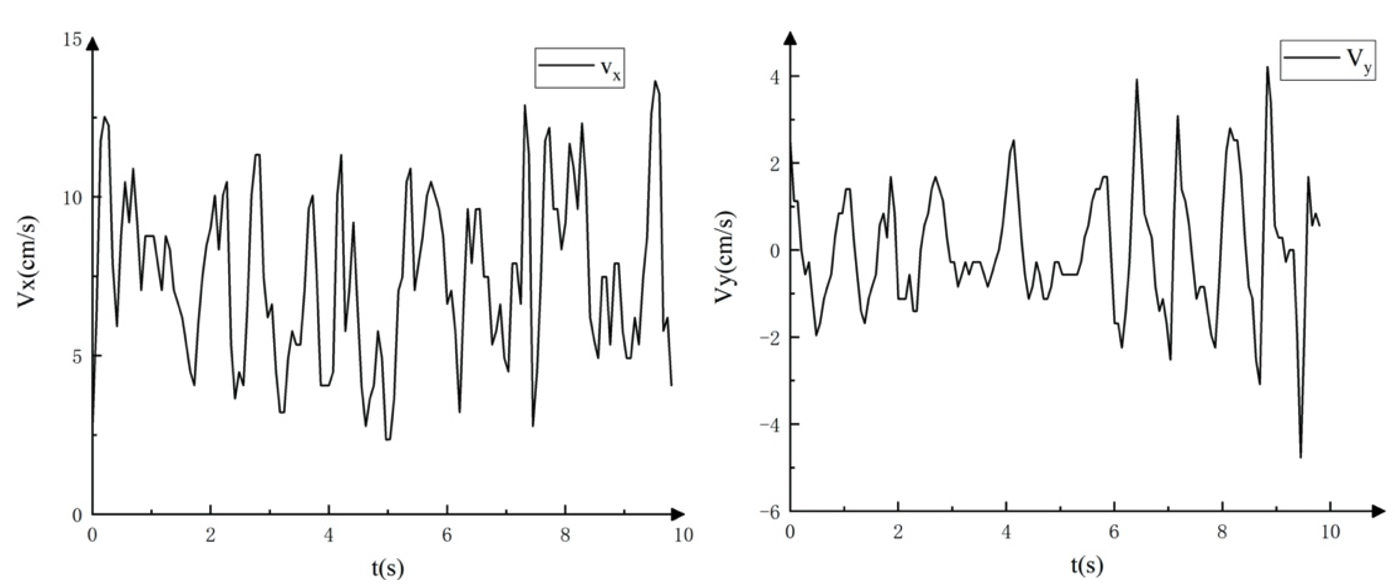

Supplement: Supplementary file 8 [file Image1.jpg]
