# Supplementary material for: Bipedal Walking of Underwater Soft Robot Based on Data-Driven Model Inspired by Octopus
Source: Front Robot AI. 2022 Apr 20;9:815435. doi: 10.3389/frobt.2022.815435 (PMC9065362; doi:10.3389/frobt.2022.815435)
Supplement: Supplementary file 4 [file Table1.docx]

# Supplementary Material

Table 1 Parameters of the soft arm

| Parameter | Value |
| --- | --- |
| Pulse width of the input signal of the servomotor $x$ | $0.5ms-2.5ms$ |
| The pulse period | $20ms$ |
| Rotation angle of the servomotor $\theta$ | $0-180^{\circ}$ |
| The radius of the servomotor turntable$r$ | $16mm$ |
| The initial distance from the fixed place of the servomotor wheel of the cable to the hole of the top of the soft arm $l_{0}$ | $15mm$ |

Table 2 Training parameters

| Parameter | Value |
| --- | --- |
| Number of training rounds | 500 |
| Maximum number of steps in a round | 100 |
| Memory space size | 20000 |
| Optimizer | RMSPropOptimizer |
| Learning rate | 0.0005 |
| Attenuation | 0.5 |
| Q network replacement parameter | 2000 |

Table 3 Reward function

| **Input**：The coordinates of the current 5 marker points of the soft arm $X_{ci}$ , the coordinates of 5 marker points of the soft arm in the last round $X_{li}$ and the target coordinates $X_{ti},i=1,2,3,4,5$. |
| --- |
| **Output**：The reward of current state $R$and the flag of termination $Terminal$ |
| 1: for sequence do  2: for episode do  3: ${Dis}_{c}=\sum_{i=1}^{5} \vert\vert X_{ci}-X_{ti}\vert\vert,{Dis}_{l}=\sum_{i=1}^{5} \vert\vert X_{li}-X_{ti}\vert\vert,$ Terminal=False  4: if step>100 \|\| Override then  5: Terminal=True  6: else if ${Dis}_{c}<\varepsilon$ then  7: Terminal=True, R=2  8: else if ${Dis}_{l}- {Dis}_{c}>0$ then  9: R=1  10: else  11: R=-1  12: end for  13: end for |

Table 4 Timing diagram of going forward

| Time\Label | 1 | 2 | 3 | 4 | 5 | 6 |
| --- | --- | --- | --- | --- | --- | --- |
| 1 | F | O | O | O | L | L |
| 2 | O | O | O | O | B | B |
| 3 | L | O | O | O | F | F |
| 4 | B | O | O | O | O | O |

Table 5 Average speed under different motion frequency

| Frequency/ Hz | Average speed/(cm/s) | Frequency/ Hz | Average speed/(cm/s) |
| --- | --- | --- | --- |
| 3.33 | 7.86 | 1.43 | 4.4 |
| 2.50 | 6.32 | 1.25 | 3.37 |
| 2.00 | 5.53 | 1.11 | 2.69 |
| 1.67 | 4.71 | 1.00 | 2.01 |
